# Monitoring mitochondrial translation by pulse SILAC

**DOI:** 10.1016/j.jbc.2022.102865

**Published:** 2023-01-02

**Authors:** Koshi Imami, Matthias Selbach, Yasushi Ishihama

**Affiliations:** 1 Graduate School of Pharmaceutical Sciences, Kyoto University, Kyoto, Japan; 2 RIKEN Center for Integrative Medical Sciences, Yokohama, Japan; 3 Max Delbrück Center for Molecular Medicine in the Helmholtz Association (MDC), Berlin, Germany; 4 Charité-Universitätsmedizin Berlin, Berlin, Germany; 5 Laboratory of Clinical and Analytical Chemistry, National Institute of Biomedical Innovation, Health and Nutrition, Osaka, Japan

**Keywords:** proteomics, pulse SILAC, translation, mitochondria, OXPHOS, protein complex, ACN, acetonitrile, AHA, azidohomoalanine, CAP, chloramphenicol, CI–V, complex I–V, DMSO, dimethyl sulfoxide, GO, Gene Ontology, HCD, higher-energy collisional dissociation, HEK293T, human embryonic kidney 293T cell line, MS, mass spectrometry, MT, mitochondrial translation, OXPHOS, oxidative phosphorylation, pSILAC, pulse stable isotope labeling of amino acids in cell culture, TMT, tandem mass tag

## Abstract

Mitochondrial ribosomes are specialized to translate the 13 membrane proteins encoded in the mitochondrial genome, which shapes the oxidative phosphorylation complexes essential for cellular energy metabolism. Despite the importance of mitochondrial translation (MT) control, it is challenging to identify and quantify the mitochondrial-encoded proteins because of their hydrophobic nature and low abundance. Here, we introduce a mass spectrometry–based proteomic method that combines biochemical isolation of mitochondria with pulse stable isotope labeling by amino acids in cell culture. Our method provides the highest protein identification rate with the shortest measurement time among currently available methods, enabling us to quantify 12 of the 13 mitochondrial-encoded proteins. We applied this method to uncover the global picture of (post-)translational regulation of both mitochondrial- and nuclear-encoded subunits of oxidative phosphorylation complexes. We found that inhibition of MT led to degradation of orphan nuclear-encoded subunits that are considered to form subcomplexes with the mitochondrial-encoded subunits. This method should be readily applicable to study MT programs in many contexts, including oxidative stress and mitochondrial disease.

In eukaryotes, both cytosolic and mitochondrial ribosomes (mitoribosomes) play a central role in protein synthesis. Cytosolic ribosomes produce constituents of the cellular proteome encoded in the nuclear genome, whereas mitoribosomes are specialized to translate the 13 membrane proteins encoded in the mitochondrial genome. These translational products are some of the subunits of oxidative phosphorylation (OXPHOS) complexes that are essential for energy generation in cells. Cytosolic and mitochondrial ribosomes coordinate their translation to enable the proper assembly of OXPHOS complexes on the inner mitochondrial membrane (1, 2, 3, 4, 5, 6, 7). In yeast, cytosolic translation controls mitochondrial translation (MT), directly facilitating balanced mitochondrial and cytosolic protein synthesis through rapid feedback between the two translation systems (1, 2, 3, 4, 6, 7). In contrast, human OXPHOS complexes appear to be synthesized proportionally to each other by cytosolic and mitochondrial ribosomes and do not rely on rapid feedback between the two translation systems (5). Thus, the balance between the mitochondrial and cytosolic translation programs is essential for maintaining mitochondrial proteostasis, that is, to prevent accumulation of unwanted and potentially harmful assembly intermediates (8). Moreover, many disease-associated mitochondrial mutations are known to impair the MT machinery (9, 10), suggesting that dysregulation of MT leads to disease.

Despite the importance of the MT system, a simple and robust method to monitor MT products is lacking. A classical approach is pulse labeling of MT products with radiolabeled amino acids, such as [^35^S]methionine and [^35^S]cysteine (11, 12), but the use of radioactive materials and the low resolution of SDS-PAGE gel-based separation of the products limits the utility of this methodology. Alternatively, mass spectrometry (MS)–based proteomic approaches have been developed to monitor protein synthesis (13). Quantitative noncanonical amino acid tagging (14, 15, 16) relies on pulse labeling of newly synthesized proteins with a methionine analog, azidohomoalanine (AHA) (17), allowing for selective enrichment of the tagged protein pool through click chemistry as well as MS-based profiling of the tagged proteins. Nascent chain proteomics using puromycin or its analogs enables isolation and identification of nascent polypeptide chains that are being elongated by the ribosomes (18, 19, 20, 21, 22, 23, 24). However, these methods require a large number of cells (typically >10^7^ cells), involve multiple steps to purify newly synthesized proteins *via* affinity purification, and/or require the isolation of ribosome complexes through density gradient ultracentrifugation.

In contrast, pulse stable isotope labeling of amino acids in cell culture (pSILAC) is a simple and robust technique for global analysis of cellular protein translation (20, 25, 26, 27, 28, 29). pSILAC involves metabolic pulse labeling of newly synthesized proteins with either heavy (*e.g.*, Arg10/Lys8) or medium–heavy (*e.g.*, Arg6/Lys4) amino acids for two cell populations of interest. The newly synthesized (labeled) proteins can be distinguished from pre-existing (nonlabeled) proteins by means of MS. The heavy to medium–heavy ratios in the MS spectra reflect the differences in protein production between the two conditions. Of note, a dynamic SILAC approach (30), a variant of SILAC that measures protein turnover by quantifying heavy (rate of synthesis) to light (rate of degradation) ratios of individual proteins over a time course, was also recently used to study the turnover rates of mitochondrial proteins in yeast (31) and humans (32, 33). Recently, the Münch’s group developed the multiplexed enhanced protein dynamics method that combines pSILAC labeling with tandem mass tag (TMT)–based multiplexing for studying stress-induced translational responses (27) and mitochondrial protein import (21). Compared with the methods described previously, pSILAC does not require many cells (from one to three orders of magnitude fewer), and the downstream experimental process is simply a conventional proteomic workflow. Thus, pSILAC would be a powerful approach to monitor MT, but its application to mitochondrial research has been limited.

One of the major challenges in the analysis of MT is that mass spectrometric identification and quantification of the 13 mitochondrial-encoded membrane proteins is hampered by poor protein identification (Fig. 1 and see the Experimental procedures section) because of the hydrophobic nature and relatively low abundance of these proteins. To overcome this problem, we present a method to comprehensively monitor protein synthesis by mitoribosomes that combines pSILAC with biochemical isolation of mitochondria. Our method offers the highest protein identification rate and the shortest MS measurement time among currently available methods. To demonstrate its utility, we applied it to examine the translational regulation of the mitochondrial- and nuclear-encoded subunits of the OXPHOS complexes.

**Figure 1:**
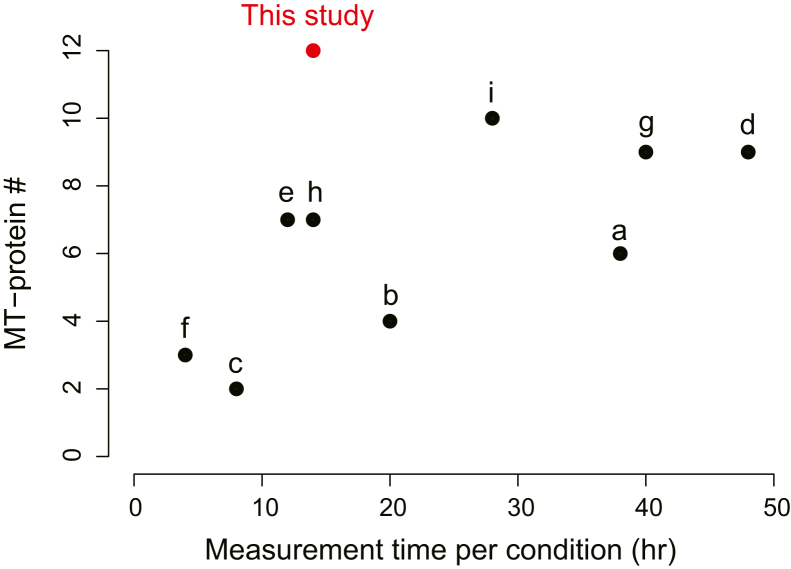
**Comparison of the present method with previous studies.** The identification numbers of MT-proteins (*left axis*) and the total LC/MS/MS measurement time (*right axis*) are shown. The present study gave the highest identification rate of MT-proteins (12 proteins) with the shortest measurement time (14 h). To compare our method with that of previous reports, we selected studies that had employed pSILAC (a (25), b (20), and c (26)), AHA (e, (16)), puromycin (f, (18), g, (22), and h (23)) or dynamic SILAC-tandem mass tag (TMT) (d (21) and i (21)). If multiple experiments were performed within a study, the single specific experiment with the highest proteome coverage was chosen. More detailed information is provided in the Experimental procedures section. AHA, azidohomoalanine; MT, mitochondrial translation; pSILAC, pulse stable isotope labeling of amino acids in cell culture.

## Results and discussion

### Biochemical optimization for comprehensive analysis of MT products

**Figure 2:**
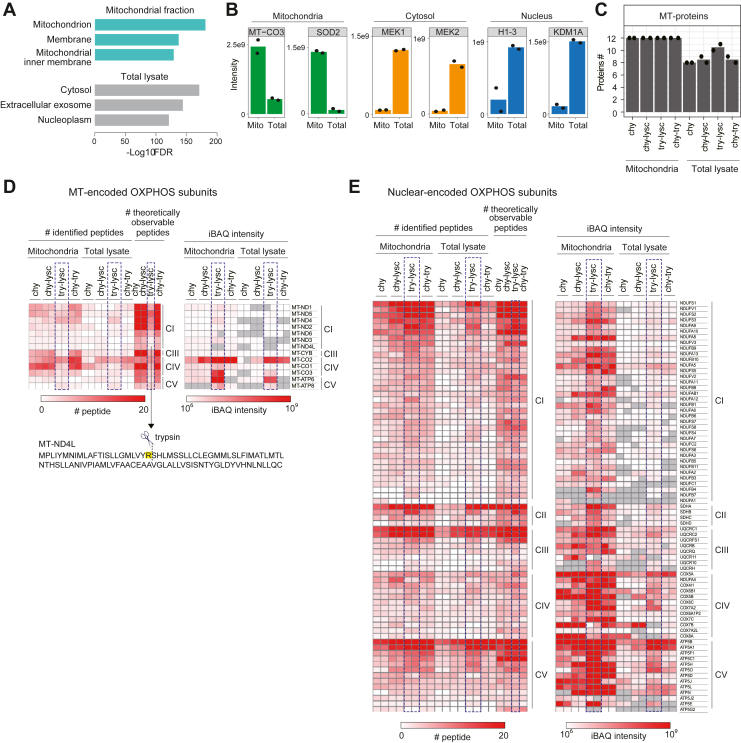
**Optimization of biochemical conditions for proteomic analysis of mitochondrial-encoded proteins.***A*, the top three Gene Ontology (GO) terms enriched in the mitochondrial fraction (*top*, *light green*) and total cell lysate (*bottom*, *gray*). *B*, abundance profiles of selected organelle markers (*left*: mitochondria, *center*: cytosol, and *right*: nucleus) for the mitochondrial fraction (Mito) and total lysate (Total). Only lysC-trypsin digestion samples are shown. *C*, the numbers of identified MT-proteins obtained under eight different conditions. The *bars* show the average number of identified proteins from two independent experiments (*filled circle*). *D* and *E*, heatmaps showing the number of identified peptides (*left*) and iBAQ values (*right*) from MT- and nuclear-encoded OXPHOS subunits. CI–V, complex I–V; iBAQ, intensity-based absolute quantification; MT, mitochondrial translation; OXPHOS, oxidative phosphorylation.

While proteomic technologies to quantify protein synthesis have been developed, comprehensive analysis of MT products (hereafter, MT-proteins or MT-encoded subunits) remains challenging, regardless of extensive peptide prefractionation and the use of high-sensitivity mass spectrometers (Fig. 1 and see the Experimental procedures section). To address this issue, we first examined whether the identification of MT-proteins could be improved by combining biochemical isolation of mitochondria with the application of combinations of proteases. Chymotrypsin cleaves on the C-terminal side of the hydrophobic essential amino acids (phenylalanine, tryptophan, and tyrosine) and thus might be suitable for digesting membrane proteins and for pSILAC, which requires essential amino acids for metabolic labeling. We adapted a reported protocol for mitochondrial isolation according to Ref. (34), as it is relatively simple (not requiring ultracentrifugation) and quick (approximately 40 min) (see the Experimental procedures section). Mitochondria pellets isolated from human embryonic kidney 293T (HEK293T) cells were lysed, and protein digestion was performed with (1) chymotrypsin, (2) chymotrypsin-lysC, (3) lysC-trypsin, or (4) chymotrypsin–trypsin. Although chymotrypsin itself was used and evaluated for mitochondrial research (33, 35), we also considered combining chymotrypsin with the other proteases. In parallel, total cell lysates were digested in the same way as a control. Two biological replicates were analyzed. A complete list of the 6442 proteins identified from total cell lysates and mitochondrial pellets is provided in Table S1. We first confirmed that mitochondrial proteins were highly enriched in the isolated mitochondrial fractions, as judged by Gene Ontology (GO) enrichment analysis (Fig 2*A* for the top three terms and Fig. S1 for all terms) and by examination of selected marker proteins (Fig. 2*B*). It should be noted that contamination with proteins from other membranes cannot be completely avoided (Fig. S1), as discussed elsewhere (36).

Figure 2*C* shows the number of MT-proteins identified by the different protocols. Isolation of mitochondria significantly enhanced the identification of MT-proteins, as compared with the total cell lysate. In every digestion protocol, 12 of the 13 MT-proteins were identified in the mitochondrial fraction, whereas on average, only nine proteins were identified in the total lysates. Importantly, it was possible to identify all 13 MT-proteins by utilizing a combination of two digestion protocols (*e.g.*, lysC-trypsin and chymotrypsin) after mitochondrial isolation, indicating that comprehensive profiling of the MT-proteins can be achieved with these two digestion protocols.

To further evaluate the relationship between protease combinations and the number of MT-proteins identified, we assessed theoretically observable (*i.e.*, a length of 6–30 amino acids) and experimentally identified peptides for both MT- and nuclear-encoded OXPHOS subunits (Figs. 2, *D* and *E* and S2). We found a clear correlation between the numbers of theoretical and identified peptides for all protease combinations (Fig. 2, *D* and *E*, *left panels*). The reason why one of the 13 MT-proteins was missed in the mitochondrial fraction in every digestion protocol (Fig. 2*C*) can be explained by observable peptides. For example, we always missed MT-ND4L when lysC-trypsin was used because there is only one arginine residue in that protein (Fig. 2*D*, *bottom*). This resulted in the production of only two tryptic peptides of 23 and 75 residues with a high content of hydrophobic amino acids, limiting the identification of MT-ND4L-derived peptides. In addition to the number of identified peptides, we assessed abundance of MT-proteins quantified from individual protocols (Figs. 2, *D* and *E* and S2, *right panels*). The different digestion protocols for a given protein should yield different numbers of peptides. To minimize and normalize this variation in the number of peptides among the different protocols, we used intensity-based absolute quantification (37) values, which are normalized intensities based on the number of observable peptides. Interestingly, even though fewer peptides were identified from MT-proteins with lysC-trypsin digestion, we observed higher intensity-based absolute quantification intensities for MT-protein-derived tryptic peptides (Fig. 2, *D* and *E*, *right panels*) compared with other protease-cleaved peptides. This observation can potentially be explained by the fact that the physicochemical properties of tryptic peptides with C-terminal positive charges enhance the solubility, ionization efficiency, and/or MS2 fragmentation efficiency of them.

Given that all the digestion protocols allowed identification of 12 MT-proteins (Fig. 2*C*), we decided to focus on lysC-trypsin digestion of isolated mitochondria for further analysis, as it produced tryptic peptides that are quantifiable with a standard pSILAC protocol using stable isotope–labeled arginine and lysine (28, 29), and also provided higher peptide intensities of MT-proteins than did other proteases. Although the chymotrypsin digestion protocols also allowed us to profile 12 MT-proteins, the broad usability of the method is hampered because a tyrosine-, phenylalanine-, and tryptophan-free custom-made medium and corresponding stable isotope–labeled amino acids are required for pulse labeling.

### pSILAC approach to monitor MT

**Figure 3:**
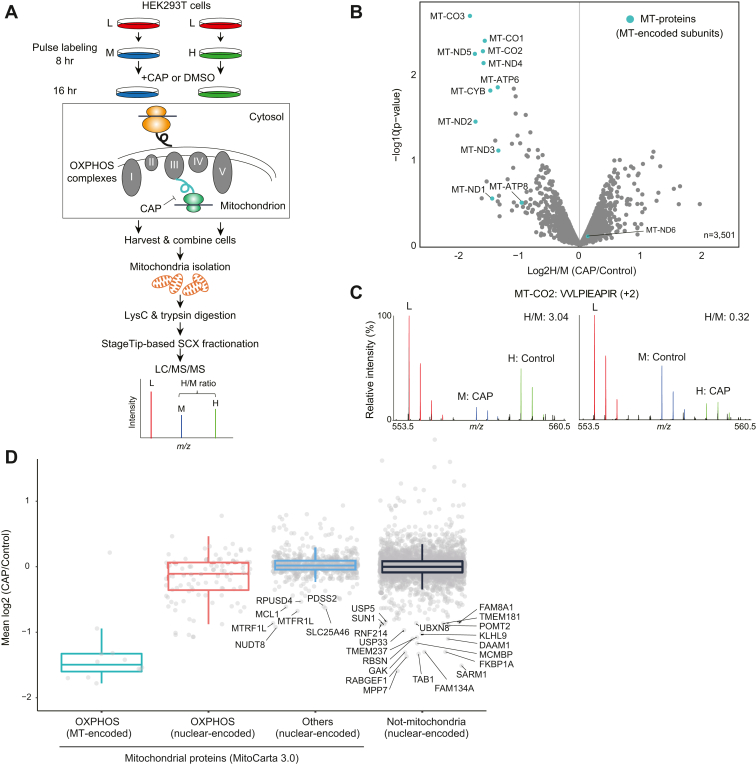
**pSILAC experiments.***A*, experimental scheme of pSILAC. HEK293T cells were pulsed-labeled with medium–heavy (M) or heavy (H) amino acids in the presence of CAP or DMSO. Two independent experiments involving a label-swap condition were performed. *B*, a volcano plot showing mean log2 fold change (CAP/DMSO) and −log10 *p* value. The MT-proteins are indicated by *light green-filled circles*. *C*, MS spectrum of an MT-CO2 peptide (VVLPIEAPIR, +2), as an example. *D*, box plots showing log2 fold change (CAP/control) of proteins grouped into four categories: MT-encoded OXPHOS subunits, nuclear-encoded OXPHOS subunits, nuclear-encoded mitochondrial proteins, and nuclear-encoded nonmitochondrial proteins. Mitochondrial proteins were defined based on MitoCarta3.0 (71). CAP, chloramphenicol; DMSO, dimethyl sulfoxide; HEK293T, human embryonic kidney 293T cell line; MT, mitochondrial translation; OXPHOS, oxidative phosphorylation; pSILAC, pulse stable isotope labeling of amino acids in cell culture.

Having established suitable biochemical conditions, we next performed pSILAC experiments. Chloramphenicol (CAP) binds to the A-site crevice on bacterial and mitochondrial ribosomes (38) and thereby inhibits mitochondrial, but not cytosolic, translation (Fig. 3*A*). We sought to assess how inhibition of MT through CAP impacts on the synthesis of both MT- and nuclear-encoded OXPHOS complex subunits by applying pSILAC methodology.

The experimental scheme of the pSILAC experiment is depicted in Figure 3*A*. HEK293T cells cultivated in “light (L)” medium were switched to “medium–heavy (M)” (^13^C_6_-arginine [Arg”6”] and D_4_-lysine [Lys”4”]) or “heavy (H)” (^13^C_6_,^15^N_4_-arginine [Arg”10”] and ^13^C_6_,^15^N_2_-lysine [Lys”8”]) medium to pulse label newly synthesized proteins. Cells were first preincubated for 8 h and then further incubated for 16 h in the presence of 10 μg/ml CAP or vehicle (dimethyl sulfoxide [DMSO]). We chose 24 h pulse labeling because this period has previously been employed in many studies (20, 25, 26, 27, 28, 29), based on the fact that newly synthesized forms (M and H) of most mammalian proteins can be detected over a period of 24 h (39), thus enabling accurate quantification of H/M ratios. Two independent experiments involving label-swap conditions were performed. For pSILAC samples, corresponding M- and H-labeled cells were combined immediately after harvesting cells to minimize technical variability during sample preparation, followed by mitochondria isolation and lysC and trypsin digestion. The digested peptides were fractionated into seven fractions using an SCX StageTip (40), and individual fractions were analyzed by means of 110 min LC/MS/MS runs with a 65 min gradient, resulting in a measurement time of approximately 14 h per condition. We then quantified the H/M ratios in MS spectra to evaluate changes in protein synthesis between CAP and DMSO treatments.

In total, we identified 4193 proteins, of which 3501 proteins were quantified in both of two independent experiments and were used for further analysis (see Table S2 for a complete list of proteins and Fig. S3 for a more detailed workflow for computational analysis). As expected, our method successfully quantified 12 of the 13 MT-proteins, all of which exhibited translational inhibition except for MT-ND6 (Fig. 3*B*). Exemplary MS spectra for an MT-CO2-derived peptide (VVLPIEAPIR, +2) are shown in Figure 3*C*. Manual inspection of the MS spectra for an MT-ND6-derived peptide (EDPIGAGALYDYGR, +2) revealed that MT-ND6 is likely to be downregulated by CAP (Fig. S4, *left panel*) as with other MT-encoded subunits. On the one hand, particular isotope peaks of M- and H-labeled MT-ND6 peptides overlapped with adjacent high-intensity peaks in one of the replicates (Fig. S4, *right panel*), thus hampering accurate quantification of H/M ratio of the peptide.

In summary, these results show that the levels of newly synthesized MT-proteins were decreased by CAP treatment (Fig. 3, *B* and *C*), confirming that our approach is indeed able to capture the expected changes in MT. The developed proteomic method provides near-comprehensive (92%) quantification of MT-proteins, representing an improvement of about twofold in protein identification as compared with previous pSILAC studies (Fig. 1 and see the Experimental procedures section).

Of note, in addition to OXPHOS subunits, amounts of several mitochondrial and nonmitochondrial proteins were modulated by CAP (Fig. 3*D* and Table S2). For example, MTRF1L (41) and RPUSD4 (42) which localize in mitochondria and positively regulate MT, were downregulated, possibly because of feedback regulation caused by the suppression of MT. We also observed the inhibition of synthesis of nonmitochondrial proteins involved in ubiquitination (USP5, MYCBP2, RABGEF1, KLHL9, RNF170, RNF214, and USP33) and membrane trafficking (GAK, RABGEF1, DBNL, SNX9, USP33, CARMIL1, PRKAA2, ARFGAP3, USE1, ARL3, RBSN, and TBC1D5), implying that these proteins may crosstalk with MT. Although the mitoribosomes appear to exclusively translate the mitochondrial mRNAs according to the ribosome profiling data (5, 43), it would be intriguing to speculate that nuclear transcripts are also regulated by the mitoribosomes.

### Relationship between MT and assembly of OXPHOS complexes

**Figure 4:**
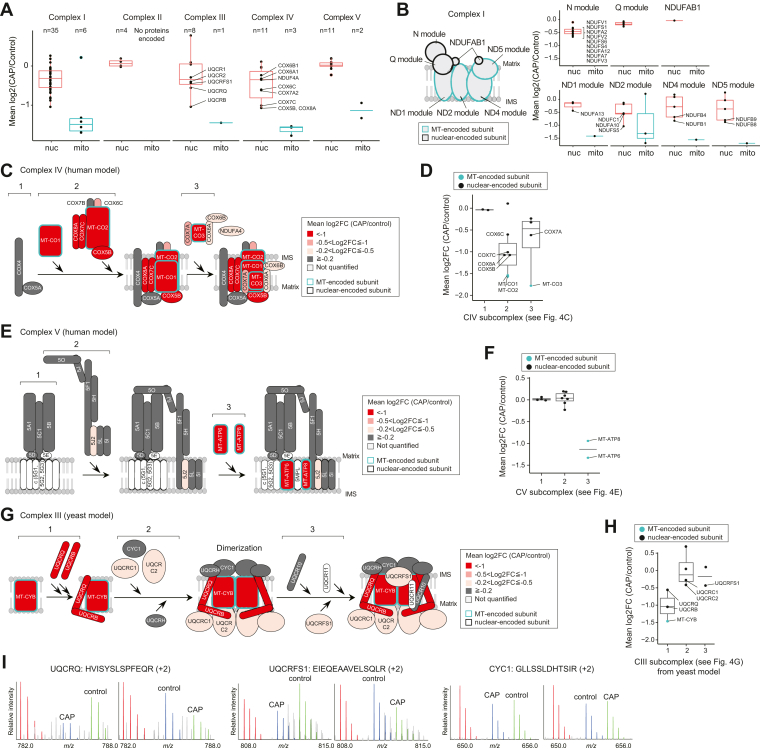
**(Post-)translational control and OXPHOS complex assembly.***A*, box plots showing log2 fold change (FC; CAP/control) of nuclear-encoded (“nuc”) and mitochondrial-encoded (“mito”) proteins in individual OXPHOS complexes. *B*, CI and its subcomplexes. *Left*, positions of subunits in CI (NADH:ubiquinone oxidoreductase) based on human data (72). *Right*, box plots showing log2 FC (CAP/control) for “nuc” and “mito” proteins. *C*, an assembly model for CIV (or cytochrome *c* oxidase) based on human data (48). Quantitative values (*i.e.*, log2 FCs [CAP/control]) are indicated by color. *D*, box plot showing log2 FCs (CAP/control) of CIV subunits according to its subcomplexes. *E*, an assembly model for CV (or ATP synthase) based on human data (45). *F*, box plots showing log2 FCs (CAP/control) of CV subunits according to its subcomplexes. *G*, a reported assembly model for CIII (cytochrome bc1 complex) based on yeast data (52). *H*, box plots showing log2 FCs (CAP/control) of CIII subunits according to its subcomplexes. *I*, exemplary MS spectra for the peptides derived from nuclear-encoded proteins (UQCRQ, UQCRFS1, and CYC1) derived peptides. CAP, chloramphenicol; CI, complex I; OXPHOS, oxidative phosphorylation.

In addition to MT-proteins, our mitochondria-focused approach afforded good coverage of OXPHOS complex subunits, including the nuclear-encoded proteins—41 of 45 proteins (91%), 4 of 4 proteins (100%), 9 of 11 proteins (82%), 14 of 21 proteins (67%), and 13 of 17 proteins (76%) from complexes I–V (CI–V), respectively. Therefore, these data can be used to assess how the nuclear-encoded subunits are (post-)translationally regulated in concert with the inhibition of MT. We found significant attenuation of the production of some nuclear-encoded subunits in CI, CIII, and CIV (Fig. 4*A*), though production of most of the nuclear-encoded OXPHOS subunits (especially CII and CV) remained unchanged.

To better understand this differential regulation of nuclear-encoded subunits, we focused on CI (NADH:ubiquinone oxidoreductase), CIV (cytochrome *c* oxidase), and CV (ATP synthase) whose assembly subcomplexes and pathways have been well characterized in humans (44, 45, 46, 47, 48). Notably, we observed a trend for nuclear-encoded subunits that are assembly partners with MT-encoded subunits to be downregulated by CAP (Fig. 4*A*). CI is composed of seven modules that are assembled individually (Fig. 4*B*, *left*). Our pSILAC data indicate that some of the nascent nuclear-encoded subunits residing in the same structural modules of the MT-encoded subunits (*i.e.*, ND1, ND2, ND4, and ND5 modules) were downregulated (Fig. 4*B*, *bottom right corner*). In contrast, N module, Q module, and NDUFAB1, which are composed of only nuclear-encoded subunits (but not MT-encoded subunits), were rather stable (Fig. 4*B*, *upper right corner*). It should be noted that N module exhibited a slight decrease compared with Q module and NDUFAB1. A recent report suggested that CI, CIII, and CIV assemble in a cooperative way (49), and CIII and CIV appear to aid the incorporation of N module into CI at the very end, indicating that N module might interact with an MT-protein(s) of CI, CIII, and CIV. Likewise, we found that the nuclear-encoded subunits (COX5B, COX6C, COX7C, and COX8A) of CIV affected by CAP are assembly partners of MT-proteins (MT-CO1, MT-CO2, and MT-CO3) (see subcomplex 2 in Fig. 4, *C* and *D*). In contrast, we observed no significant regulation of the early assembly subunits (COX4 and COX5A) that do not form a subcomplex with MT-proteins (see subcomplex 1 in Fig. 4, *C* and *D*). Similar results were obtained for CV (Fig. 4, *E* and *F*); MT-ATP6 and MT-ATP8 are involved in the late step of the complex assembly (step 3 in Fig. 4, *E* and *F*), and therefore, it is reasonable that the early and intermediate subcomplexes of the nuclear-encoded subunits were not regulated (see subcomplexes 1 and 2 in Fig. 4, *E* and *F*).

These results support the notion that “orphan” subunits (*i.e.*, proteins that cannot be assembled into multiprotein complexes) are subject to (post-)translational degradation (8, 50, 51) or a translational pause (4) to prevent accumulation of unwanted assembly intermediates. Consistent with this idea, we observed a trend that nuclear-encoded proteins are coregulated with MT-proteins within the same subcomplex (Fig. 4, *B*, *D* and *F*). Our data clearly indicate that abundance of nuclear-encoded subunits decreases when they lose a partner MT-encoded subunit within the same subcomplex module. In other words, whether nuclear-encoded subunits form subcomplexes with MT-encoded subunits is likely to be inferred based on changes in the levels of newly synthesized subunits (*i.e.*, H/M ratios) induced by CAP treatment.

### Inferring members of intermediate subcomplexes of CIII

We next sought to infer the intermediate steps (subcomplexes) of CIII (cytochrome bc1 complex) assembly because so far only the first and last steps of its assembly are well understood in humans (46, 52). The assembly model of CIII from yeast (53, 54, 55) is shown in Figure 4*G*. In accordance with the initial step, inhibiting the translation of MT-CYB led to (post-)translational repression of its partner subunits, UQCRQ and UQCRB (see subcomplex 1 in Fig. 4, *G*–*I*, *left*). Furthermore, consistent with the last step of the assembly in which the Rieske Fe–S protein, UQCRFS1, joins the pre-CIII assembly (46), orphan UQCRFS1 was downregulated (see subcomplex 3 in Fig. 4, *G*–*I*, middle for exemplary MS spectra for UQCRFS1). Intriguingly, in contrast to the yeast model, our results imply that UQCRC1 and UQCRC2 may be incorporated into the early assembled complex of MT-CYB, UQCRQ, and UQCRB (see subcomplex 1 in Fig. 4, *G* and *H*) during the initial/intermediate steps because the abundances of newly synthesized UQCRC1 and UQCRC2 decreased in concert with the translational inhibition of MT-CYB (Fig. 4, *G* and *H*). In contrast, other subunits (CYC1, UQCRH, and UQCR10) remained unchanged, indicating that these subunits do not form subcomplexes with MT-CYB and might form distinct module(s) (see Fig. 4*I*, *right* for exemplary MS spectra for CYC1).

### Orphan newly synthesized subunits are more quickly degraded than nonorphan subunits

**Figure 5:**
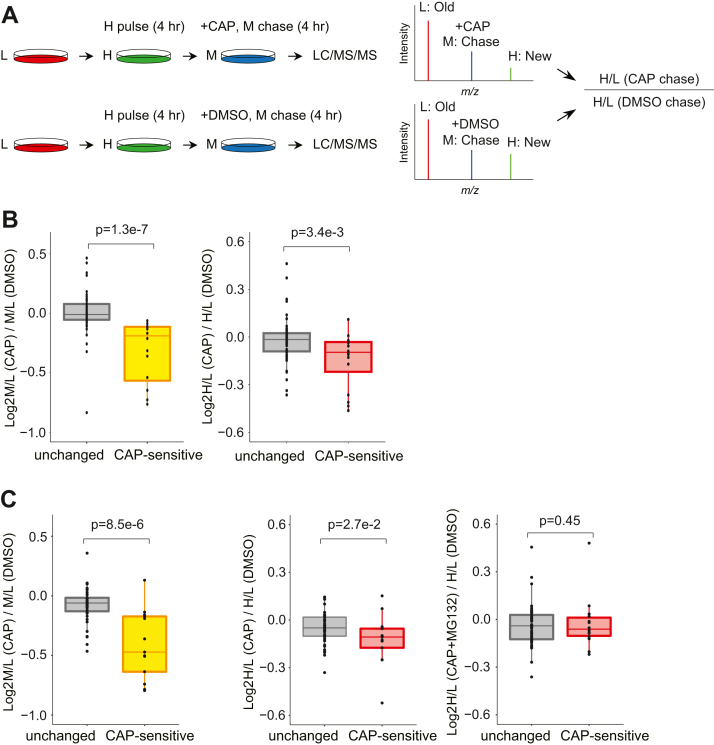
**Orphan nuclear-encoded OXPHOS subunits are degraded after protein synthesis.***A*, experimental scheme of a global pulse-chase experiment. HEK293T cells were pulse-labeled with H amino acids for 4 h, followed by chasing newly synthesized H forms for 4 h by switching to medium containing M amino acids in the presence of CAP or DMSO. The degree of degradation of newly synthesized H proteins can be assessed based on the H/L (CAP)/H/L (DMSO) ratios. *B*, effects of CAP on protein synthesis and degradation of nuclear-encoded subunits. A box plot showing the degree of inhibition of protein synthesis of newly made M forms between CAP and DMSO treatments (*left panel*). A box plot showing the degree to which newly made H forms are degraded with CAP treatment (*right panel*). “Unchanged” and “CAP-sensitive” represent nuclear-encoded subunits whose protein synthesis was unchanged and inhibited by CAP, respectively (Fig. 4). *p* Values were computed using the one-sided Wilcoxon rank-sum test. *C*, same as in (*B*) but showing results from MG132 treatment. The contribution of degradation of newly synthesized H proteins can be assessed based on the H/L (CAP)/H/L (DMSO) *versus* H/L (CAP + MG132)/H/L (DMSO). *p* Value was computed using the one-sided Wilcoxon rank-sum test. CAP, chloramphenicol; DMSO, dimethyl sulfoxide; HEK293T, human embryonic kidney 293T cell line; OXPHOS, oxidative phosphorylation.

The data (Fig. 4) presented so far indicate that orphan nuclear-encoded OXPHOS subunits whose partner MT-encoded subunits are lost are more likely to be degraded as seen in other protein complexes (50). To confirm this, we performed a slightly modified version of previously reported global pulse-chase experiments (50); HEK293T cells were pulse-labeled with H amino acids for 4 h, followed by chasing newly synthesized H forms for another 4 h by switching to medium containing M amino acids in the presence of CAP or DMSO (Fig. 5*A*). If newly synthesized (H) proteins are less stable than old (L) proteins, their H/L ratios are expected to decrease during the chase. Hence, this experiment should allow us to assess the extent of degradation of newly synthesized proteins during CAP chase by computing the H/L (CAP)/H/L (DMSO) ratios. To this end, we grouped the nuclear-encoded subunits into the two categories; “unchanged (log2 H/M ≧ −0.5)” or “CAP-sensitive (log2 H/M < −0.5),” according to the pSILAC experiment (Figs. 3 and 4) and asked whether proteins in the CAP-sensitive group (*i.e.*, orphan subunits) are less stable than those in the unchanged group. We first confirmed that CAP inhibited protein synthesis (M-channel) of the CAP-sensitive group more effectively than that of the unchanged group (Fig. 5*B*, *left panel* and Table S3). Consistent with our hypothesis, we observed a trend that newly made subunits (H-channel) in the CAP-sensitive group were less stable than the other subunits (Fig. 5*B*, *right panel*). We also repeated the pulse-chase experiment and observed the same trend (Fig. S5).

To further assess if the nuclear-encoded orphan subunits are degraded *via* the proteasome, we performed an additional experiment where cells were treated with the proteasome inhibitor MG132 during the CAP chase. To do this, HEK293T cells were pulse-labeled with H amino acids for 4 h, followed by chasing newly synthesized H forms for another 4 h in a medium containing M amino acids in the presence of CAP and MG132. We also repeated the same experiments shown in Figure 5*A* and recapitulated the previous findings (Fig. 5*C*, *left and middle panels*). While newly made subunits (H-channel) in the CAP-sensitive group were less stable than the other subunits (Fig. 5*C*, *middle panel*), consistent with Figures 5*B* and S5, this trend was not seen in the MG132-treated cells (Fig. 5*C*, *right panel*). Thus, proteasome inhibition indeed attenuated the degradation of the nuclear-encoded subunits, indicating that these proteins are degraded through the proteasome. This result also supports a recent finding showing that the turnover of several OXPHOS proteins is dependent upon the ubiquitin–proteasome system (56).

Collectively, these results suggest that protein degradation is a key pathway of elimination of orphan nuclear-encoded subunits that cannot form a subcomplex with MT-encoded subunits.

### Validation of the method using normal diploid human cells

**Figure 6:**
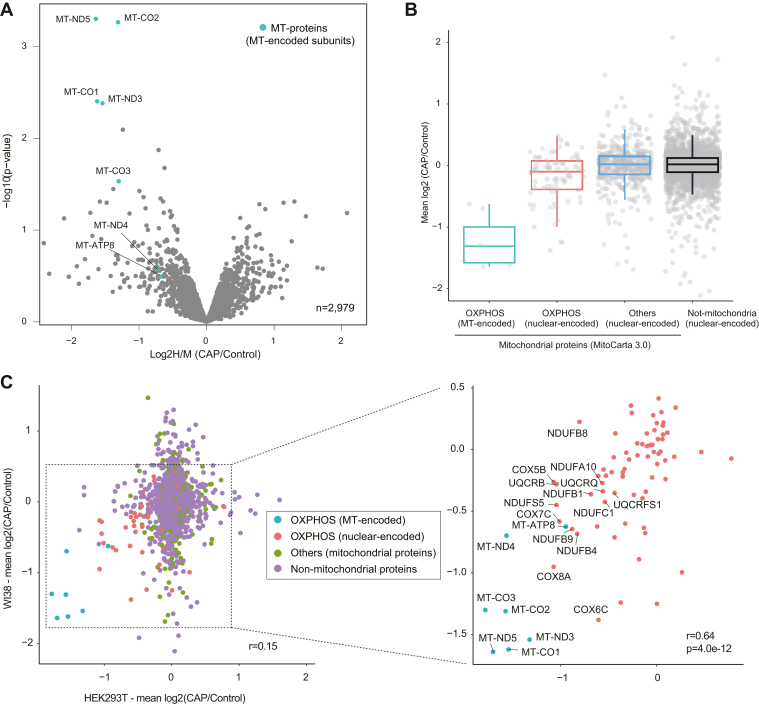
**Validation of mitochondrial pSILAC experiments using WI38 cells.***A*, a volcano plot showing mean log2 fold change (CAP/DMSO) and −log10 *p* value for quantified proteins. The MT-encoded subunits are indicated by *light green-filled circles*. Three independent experiments were performed: In the first and second independent experiments, WI38 cells were pulsed-labeled with medium–heavy (M) or heavy (H) amino acids in the presence of CAP or DMSO, respectively, whereas in the third experiment, M- and H-labeled cells were treated with DMSO or CAP, respectively. *B*, box plots showing log2 fold change (CAP/control) of proteins grouped into four categories: MT-encoded OXPHOS subunits, nuclear-encoded OXPHOS subunits, nuclear-encoded mitochondrial proteins, and nuclear-encoded nonmitochondrial proteins. *C*, scatter plots showing correlations of log2 fold change (CAP/control) of proteins between HEK293T and WI38 cells. The four categories of proteins are color labeled. CAP, chloramphenicol; DMSO, dimethyl sulfoxide; HEK293T.human embryonic kidney 293T cell line; MT, mitochondrial translation; OXPHOS, oxidative phosphorylation; pSILAC, pulse stable isotope labeling of amino acids in cell culture.

Finally, we sought to validate our method and results using WI38 cells, which are normal diploid human fibroblasts derived from lung tissue. Three independent pSILAC experiments were done with WI38 cells, and results from an analysis of the isolated mitochondria confirmed the key findings from HEK293T cells. First, we found that CAP treatment also specifically inhibited translation of the MT-encoded proteins (Fig. 6*A*). Although we only identified eight MT-encoded proteins, of which seven proteins were quantified in all three experiments, this may be due to the lower protein expression level of particular MT-encoded proteins in WI38 compared with HEK293T cells. Indeed, only nine MT-proteins could be identified in a deep proteomic study of WI38 cells where nearly 10,000 proteins were identified (57). Second, a specific subset of nuclear-encoded OXPHOS subunits was also found to be coregulated with MT-encoded subunits (Fig. 6, *B* and *C*). Notably, the changes in the levels of newly synthesized OXPHOS subunits were significantly correlated between WI38 and HEK293T cells (Pearson correlation coefficient *r* = 0.64), whereas other mitochondrial and nonmitochondrial proteins did not show such strong correlation (Fig. 6*C*). Importantly, specific nuclear-encoded subunits that showed CAP-induced reduction in their abundance in HEK293T were also downregulated in WI38 cells, recapitulating the observations shown in Figure 4. Collectively, these data indicate that the (post-)translational control of OXPHOS multiprotein complexes is partially cell type independent.

## Conclusions

While pSILAC is an established approach for studying protein synthesis, its application to MT has been limited. The significance of this study lies in the improvement and development of the pSILAC approach combined with a simple biochemical separation for mitochondria. To our knowledge, this is the first study to achieve a near-comprehensive profiling of nascent MT-proteins translated by mitoribosomes. Moreover, this methodology provides a global view of OXPHOS complex assembly on the basis of (post-)translational regulation of mitochondrial- and nuclear-encoded proteins. We found that CAP-mediated inhibition of MT induced degradation of the nuclear-encoded proteins of OXPHOS complexes, and this regulation appears to be maintained at the structural module level; our results suggest that orphan nascent nuclear-encoded proteins are degraded in concert with the loss of their partner MT-proteins in the same structural module. Very recently, a pSILAC-TMT approach revealed that the protein import into mitochondria is regulated at the levels of cytosolic translation and protein uptake during stress (21). Also, it was shown by ribosome profiling that human OXPHOS complexes are synthesized proportionally to each other by cytosolic and mitochondrial ribosomes (5). In addition to our findings, these exciting results obtained by cutting-edge technologies will facilitate our understanding of mitochondrial proteostasis; how mitochondrial proteome is shaped through the two translation systems, the mitochondrial protein import systems, and the protein degradation systems. We believe that this methodology will enable us to probe MT programs in many contexts, including oxidative stress and mitochondrial disease.

### Limitations of the study

It is challenging to isolate genuine organelle residents biochemically without contaminants. Here, we adapted a relatively simple protocol for mitochondria isolation that allowed us to enrich mitochondrial proteins (Figs. 2*A* and S1), but nevertheless, contaminants from other organelles (*e.g.*, endoplasmic reticulum) were observed. A method for preparation of highly purified mitochondria combined with density gradient centrifugation or affinity purification may help to improve readouts of pSILAC experiments, such as identification of mitochondrial proteins/peptides, although there is always a tradeoff between the required time for preparation and the purity of the organelle. Our mitochondrial purification and digestion protocol always missed one specific MT-encoded protein (Fig. 2*C*). Thus, a combination of two digestion protocols (*e.g.*, lysC-trypsin and chymotrypsin) would be required for comprehensive profiling of the 13 MT-proteins. Also, our pSILAC experimental set-up is specialized to monitor changes in protein production of MT-proteins by quantifying metabolically labeled proteins under two conditions. An alternative pSILAC set-up described elsewhere (39, 58) might make a useful combination to quantify protein degradation, synthesis, and turnover rates.

## Experimental procedures

### Cell culture and pulse labeling

HEK293T and WI38 cells obtained from American Type Culture Collection were cultured in Dulbecco's modified Eagle's medium (Fujifilm Wako) containing 10% fetal bovine serum (Thermo Fisher Scientific) in 10 cm diameter dishes. All cells were maintained in a humidified 37 °C incubator with 5% CO_2_. For pulse SILAC labeling (related to Figs. 3 and 6), the cell culture medium was switched to arginine- and lysine-free Dulbecco's modified Eagle's medium (Thermo Fisher Scientific) supplemented with 10% fetal bovine serum and either “heavy” amino acids [0.398 mM l-(^13^C_6_,^15^N_4_)-arginine (Arg”10”) and 0.798 mM l-(^13^C_6_,^15^N_2_)-lysine (Lys”8”)] or “medium–heavy” amino acids [0.398 mM l-(^13^C_6_)-arginine (Arg”6”) and 0.798 mM l-(D_4_)-lysine (Lys”4”)] (Cambridge Isotope Laboratories). For CAP (Fujifilm Wako) treatment, cells were first preincubated with the corresponding SILAC medium for 8 h and then further incubated for 16 h in the presence of 10 μg/ml CAP or vehicle (DMSO). The cells were washed and harvested in ice-cold PBS and pelleted by centrifugation at 600*g* for 5 min at 4 °C. Label-swap (biological duplicate) experiments were performed. For global pulse-chase experiments (related to Fig. 5), cells were pulse-labeled with “heavy” amino acids for 4 h as described previously, followed by chasing newly synthesized “heavy” forms for 4 h by switching to medium containing “medium–heavy” amino acids in the presence of 10 μg/ml CAP (Figs. 5*B* and S5) or 10 μg/ml CAP and 10 μM MG132 (Fig. 5*C*). Two independent experiments of the pulse-chase experiments under the CAP condition were performed. As a vehicle control, cells were chased in the presence of 0.1% DMSO instead of CAP and used as a universal reference.

### Mitochondria isolation

Mitochondria were isolated from five million cells in a 10 cm dish as reported (34). For pSILAC samples, corresponding medium–heavy and heavy-labeled cells were combined at this stage. The cell pellets were resuspended in 500 μl of ice-cold mitochondria isolation buffer (10 mM Tris–Mops [pH 7.4] containing 1 mM EGTA/Tris and 200 mM sucrose). The cells were homogenized using a glass/Teflon Potter Elvehjem homogenizer (2 ml volume; 40 strokes). The homogenate was transferred to a new 1.5 ml tube and centrifuged at 600*g* for 10 min at 4 °C. The supernatant was transferred to a new 1.5 ml tube and centrifuged at 7000*g* for 10 min at 4 °C. The pellet containing mitochondria was resuspended in 100 μl of ice-cold mitochondria isolation buffer and centrifuged at 7000*g* for 10 min at 4 °C. The supernatant was discarded, and the pellet was used as the mitochondrial fraction. Approximately 80 and 50 μg protein were obtained from mitochondrial pellets of HEK293T and WI38, respectively.

### Protein digestion

Protein digestion was performed according to the phase-transfer surfactant–aided digestion protocol, as described previously (59, 60). Briefly, the mitochondrial fraction was lysed with phase-transfer surfactant buffer (12 mM sodium deoxycholate [Fujifilm Wako], 12 mM sodium *N*-lauroyl sarcosinate [Fujifilm Wako] in 0.1 M Tris–HCl [pH 8.0]) and incubated with 10 mM DTT at 37 °C for 30 min, followed by alkylation with 50 mM iodoacetamide at 37 °C for 30 min in the dark. The samples were diluted five times with 50 mM ammonium bicarbonate. To optimize the digestion protocol for MT products, proteins were digested with (1) chymotrypsin (Promega), (2) chymotrypsin and lysyl endopeptidase (lys-C) (Fujifilm Wako), (3) lys-C and trypsin (Promega), and (4) chymotrypsin and trypsin at a protein-to-protease ratio of 50:1 (w/w) overnight at 37 °C on a shaking incubator. For the pSILAC experiments, proteins were first digested with lys-C for 3 h at 37 °C and then with trypsin overnight at 37 °C on a shaking incubator. Next day, an equal volume of ethyl acetate (Fujifilm Wako) was added to the sample, and digestion was quenched by adding 0.5% TFA (final concentration). The samples were shaken for 1 min and centrifuged at 16,000*g* for 2 min at 25 °C. The organic phase containing sodium deoxycholate and sodium *N*-lauroyl sarcosinate was discarded. The resulting peptide solution was evaporated in a SpeedVac, and the residue was resuspended in 200 μl 0.1% TFA and 5% acetonitrile (ACN). The peptides corresponding to 10 to 20 μg protein were desalted with an SDB-XC StageTip (61) or fractionated into seven fractions using an SDB-XC-SCX StageTip (40). Each sample solution was evaporated in a SpeedVac, and the residue was resuspended in 0.5% TFA and 4% ACN. A peptide sample corresponding to 500 ng protein was injected into MS.

### LC/MS/MS analysis

Nano-scale reversed-phase liquid chromatography coupled with tandem MS (nanoLC/MS/MS) was performed on an Orbitrap Fusion Lumos mass spectrometer (Thermo Fisher Scientific), connected to a Thermo Ultimate 3000 RSLCnano pump and an HTC-PAL autosampler (CTC Analytics) equipped with a self-pulled analytical column (150 mm length × 100 μm i.d.) (62) packed with ReproSil-Pur C18-AQ materials (3 μm; Dr Maisch GmbH). The mobile phases consisted of (A) 0.5% acetic acid and (B) 0.5% acetic acid and 80% ACN. For pSILAC experiments using HEK293T (Fig. 3), peptides were eluted from the analytical column at a flow rate of 500 nl/min by altering the gradient: 5 to 10% B in 5 min, 10 to 40% B in 60 min, 40 to 99% B in 5 min and 99% for 5 min, and a 300 min gradient was used for the biochemical optimization. The Orbitrap Fusion Lumos instrument was operated in the data-dependent mode with a full scan in the Orbitrap followed by MS/MS scans for 3 s using higher-energy collisional dissociation (HCD). The applied voltage for ionization was 2.4 kV. The full scans were performed with a resolution of 120,000, a target value of 4 × 10^5^ ions, and a maximum injection time of 50 ms. The MS scan range was *m/z* 300 to 1500. The MS/MS scans were performed with 15,000 resolution, 5 × 10^4^ target value, and 50 ms maximum injection time. The isolation window was set to 1.6, and the normalized HCD collision energy was 30. Dynamic exclusion was applied for 20 s. For the pSILAC experiments using WI38 cells (Fig. 6), nanoLC/MS/MS was performed on an Orbitrap Exploris 480 mass spectrometer (Thermo Fisher Scientific). The mass range of the survey scan was from 300 to 1500 *m/z* with a resolution of 60,000, 300% normalized automatic gain control target, and auto maximum injection time. The first mass of the MS/MS scan was set to 120 *m/z* with a resolution of 15,000, standard automatic gain control, and auto maximum injection time. Fragmentation was performed by HCD with a normalized collision energy of 30%. The dynamic exclusion time was set to 20 s.

### Database searching and protein quantification

All raw files were analyzed and processed by MaxQuant (version 1.6.0.13 or 1.6.15.0) (63). Search parameters included two missed cleavage sites and variable modifications such as methionine oxidation, protein N-terminal acetylation, and SILAC-specific modifications [L-(^13^C_6_,^15^N_4_)-arginine, L-(^13^C_6_,^15^N_2_)-lysine, L-(^13^C_6_)-arginine, and L-(D_4_)-lysine]. Cysteine carbamidomethylation was set as a fixed modification. The peptide mass tolerance was 4.5 ppm, and the MS/MS tolerance was 20 ppm. The database search was performed with Andromeda (64) against the UniProt/Swiss-Prot human database (downloaded on October 2014) with common serum contaminants and enzyme sequences. The false discovery rate was set to 1% at the peptide spectrum match level and protein level. The “match between runs” functions were employed. For protein quantification, a minimum of one unique peptide ion was used, and to ensure accurate quantification, we required proteins to be quantified in all samples for further analysis. Protein intensities from SILAC medium–heavy and heavy channels were normalized using the variance stabilization normalization (65) in the R package of DEP (66) to correct for mixing error between the two SILAC-labeled lysates. *p* Values were computed based on differential expression of proteins using protein-wise linear models and empirical Bayes statistics through the *limma* function (67).

### Assessment of the number of identified MT products and measurement time (related to Fig. 1)

There are a number of studies on cellular translation using pSILAC, AHA, and/or puromycin, as described in the introduction section. To compare our findings with those of previous reports, we chose studies that had employed pSILAC with medium–heavy and heavy amino acids (*i.e*., triplex SILAC) and that showed reasonably high proteome coverage (Table 1). Recent studies using AHA, puromycin (and its analog), or dynamic SILAC-TMT were also included. If multiple experiments were performed within a study, the single specific experiment with the highest proteome coverage was chosen (Fig. 1). The measurement time per experiment is a conservative estimate, as some studies provided only the LC gradient time and did not mention total measurement time.

**Table 1:** Method comparison

Reference	This study	a. (25)	b. (20)	c. (26)	d. (27)	e. (16)	f. (18)	g. (22)	h. (23)	i. (21)
PubMed ID	—	24637697	26183718	30220558	31812349	27764671	23934657	32531160	32926143	34847359
Measurement time (h)	14	38	20	8	48	12	4	40	14	28
No. of MT-proteins	12	6	4	2	9	7	3	9	7	10
No. of total proteins	3501	5435	5126	3422	6039	5940	2535	2589	4365	4074
Methodology	pSILAC	pSILAC	pSILAC	pSILAC	pSILAC-TMT	AHA	Puro	Puro-TMT	Puro-pSILAC	TMT-pSILAC
Cell type	HEK293T	HeLa	SW480	HEK293T	HeLa	Mouse neurons	HeLa	THP-1	HeLa	HeLa
MS	Fusion Lumos	LTQ-Orbitrap	LTQ-Orbitrap Elite	Q-Exactive Plus	Fusion Lumos	Q-Exactive Plus	Q-Exactive	LTQ-Orbitrap Elite	Fusion Lumos	Fusion Lumos
Fractionation	StageTip-based SCX fractionation (7 fractions)	GeLCMS (15 slices)	GeLCMS (20 slices)	Single shot	High pH-reversed phase fractionation (24 fractions)	Single shots (4 times)	Single shot	High pH-reversed phase fractionation (20 fractions)	StageTip-based SCX fractionation (7 fractions)	High pH-reversed phase fractionation (8 fractions)

### GO enrichment analysis (related to Fig. 2*A*)

GO enrichment analysis was performed using DAVID (The Laboratory of Human Retrovirology and Immunoinformatics) (68). To identify enriched GO terms in the mitochondrial fraction and total cell lysate, we used only proteins quantified in all the digestion protocols. The top three enriched terms for cellular components are shown in Figure 2*A*, whereas a full list of enriched GO terms is shown in Fig. S1. False discovery rates were corrected by the Benjamini–Hochberg method.

### Analysis of global pulse-chase experiment (related to Fig. 5)

Only nuclear-encoded OXPHOS subunits were analyzed, and they were grouped into the two categories, “unchanged (log2 H/M ≧ −0.5)” or “CAP-sensitive (log2 H/M < −0.5),” based on the pSILAC experiment shown in Figures 3 and 4. Heavy (H) peaks in MS spectra represent the abundance of newly synthesized proteins after the chase in the presence of CAP or DMSO. Light (L) peaks indicate pre-existing proteins. Hence, the degree of degradation of newly synthesized H proteins induced by CAP treatment can be assessed by computing H/L (CAP)/H/L (DMSO). Individual dots in the box plots (Figs. 5*B* and S5) represent log2 (H/L [CAP]/H/L [DMSO]) values for individual nuclear-encoded subunits categorized into the “unchanged” or “CAP-sensitive” group. The *p* values were computed using the one-sided Wilcoxon rank-sum test.

## Data availability

The proteomics data have been deposited to the ProteomeXchange Consortium *via* jPOST (69, 70) partner repository with the dataset identifier JPST001007 (PXD022476 for ProteomeXchange).

## Supporting information

This article contains supporting information (65).

## Conflict of interest

The authors declare that they have no conflicts of interest with the contents of this article.
