# Supplementary material for: Monitoring mitochondrial translation by pulse SILAC
Source: J Biol Chem. 2023 Jan 2;299(2):102865. doi: 10.1016/j.jbc.2022.102865 (PMC9922817; doi:10.1016/j.jbc.2022.102865)
Supplement: Supplemental Figures S1–S5 [file mmc1.pdf]

Supporting Information

Monitoring mitochondrial translation by pulse SILAC

Koshi Imami1, 2*, Matthias Selbach3, 4, Yasushi Ishihama1,5*

1 Graduate School of Pharmaceutical Sciences, Kyoto University, 606-8501 Kyoto, Japan
2 RIKEN Center for Integrative Medical Sciences, 230-0045 Yokohama, Japan
3 Max Delbrück Center for Molecular Medicine in the Helmholtz Association (MDC), 13125 Berlin, Germany
4 Charité-Universitätsmedizin Berlin, 10117 Berlin, Germany
5 Laboratory of Clinical and Analytical Chemistry, National Institute of Biomedical Innovation, Health and Nutrition, 567-0085 Osaka, Japan

*Corresponding authors:
Koshi Imami (Lead contact)
RIKEN Center for Integrative Medical Sciences,
Tel.: +81-45-503-9696
email: koshi.imami@gmail.com

Yasushi Ishihama
Graduate School of Pharmaceutical Sciences, Kyoto University
Tel.: +81-75-753-4555
Fax.: +81-75-753-4601
email: yishiham@pharm.kyoto-u.ac.jp

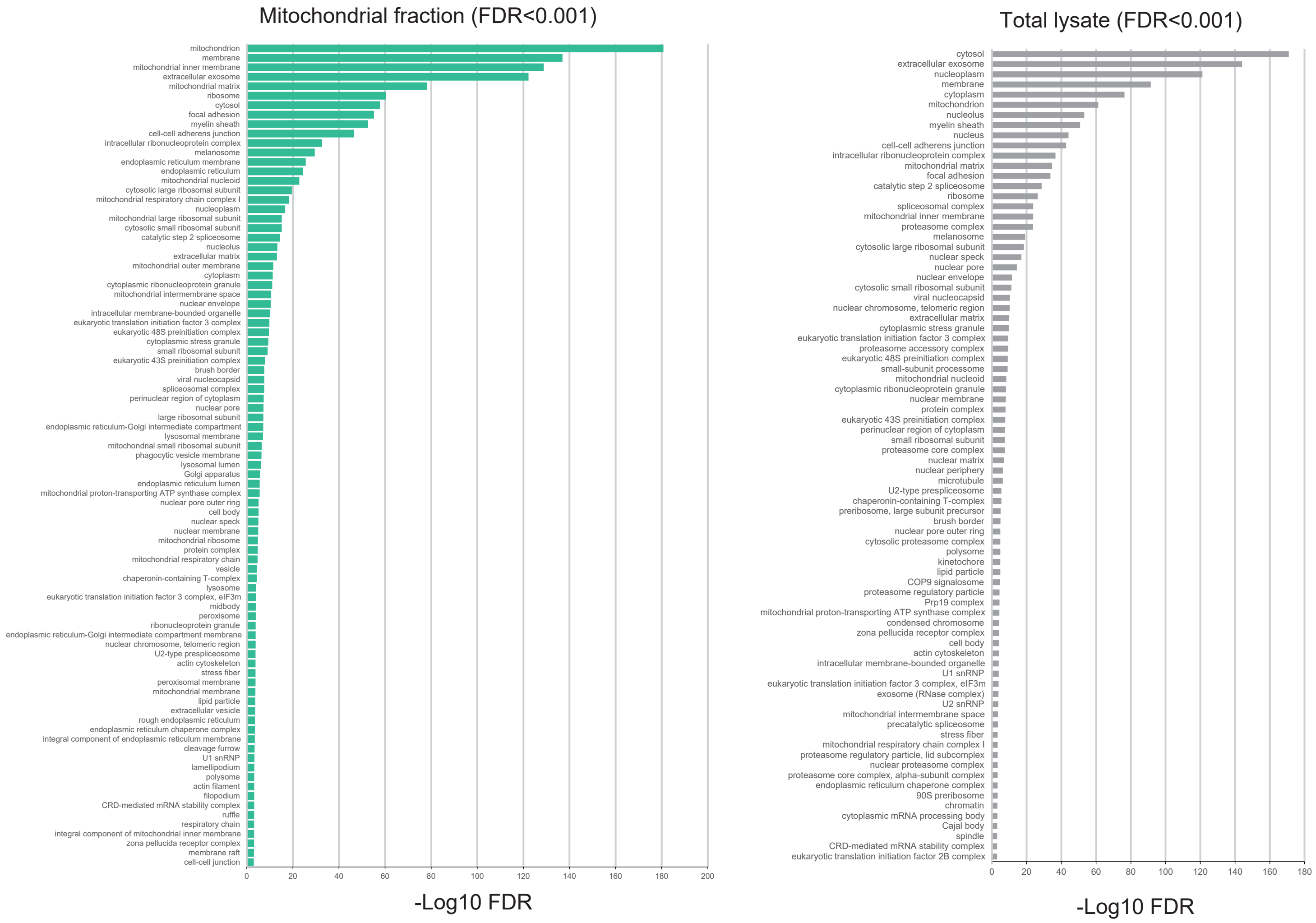

Figure S1: Enriched GO terms in mitochondrial fraction- (left) and total lysate- (right) derived samples, related to Figure 2A.

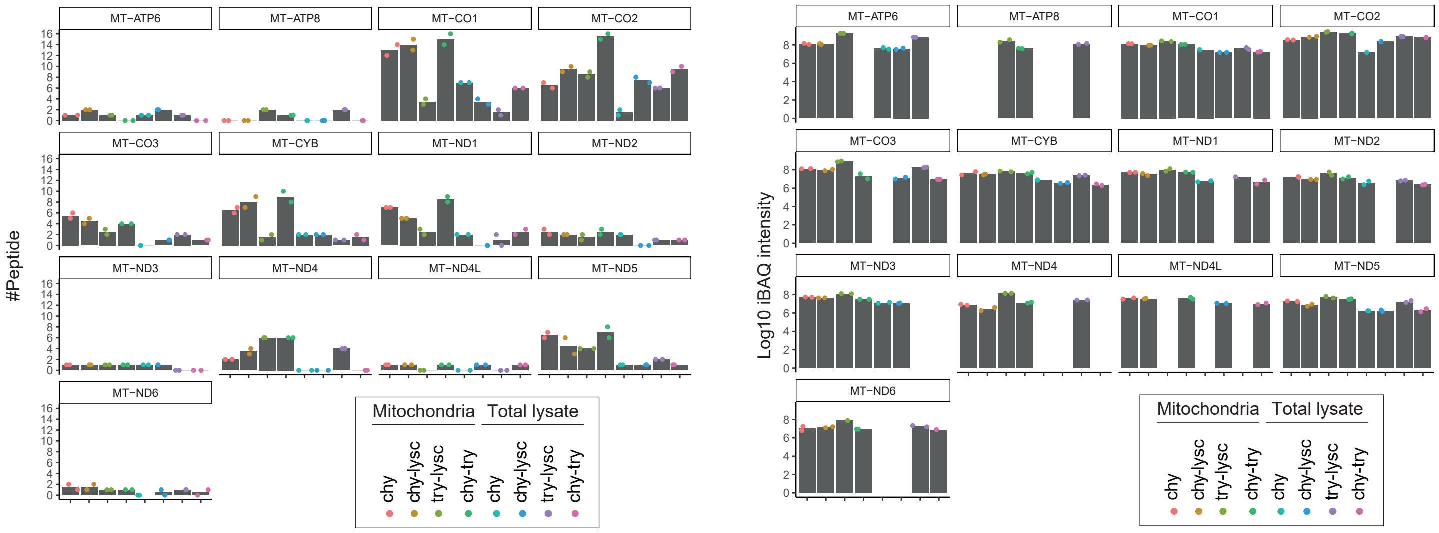

Figure S2: The number of identified MT-proteins (left) and thier iBAQ intensities (right) obtained under 8 different conditions, related to Figure 2D.

The bars show the average number of identified proteins or their log10 iBAQ intensities from two independent experiments (filled-circle).

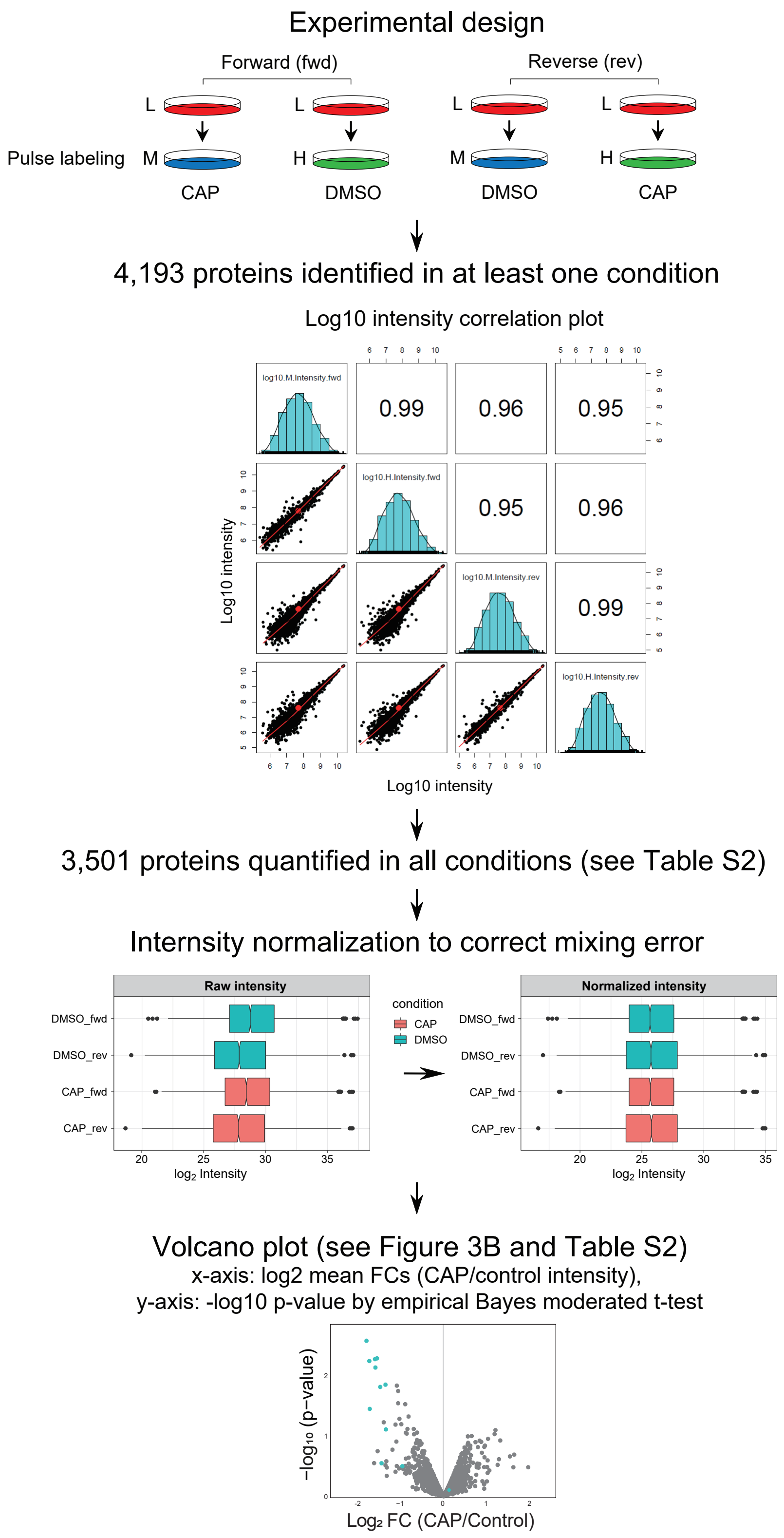

Figure S3: A workflow for the analysis of the pSILAC data related to Figures 3 and 4.

Two independent pSILAC experiments (forward and reverse label-swap) were performed; of the 4,193 proteins identified under at least one condition, 3,501 proteins quantified under all conditions were used for the following analysis. To correct the mixing ratio of H and M samples, variance stabilization normalization (Huber et al. 2002) was performed. Finally, mean log2FC and t-test were used to obtain a volcano plot (Figure 3B)

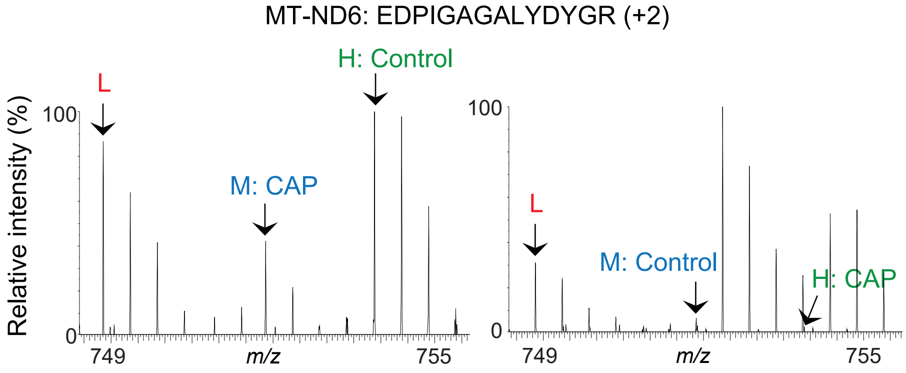

Figure S4: Manual inspection of MS spectra of an MT-ND6-derived peptide, related to Figure 3B. The expected translational inhibition of MT-ND6 was observed in the left panel (M: CAP, H: control), in contrast to the output result where translation of MT-ND6 was not apprently inhibited (see Figure 3B). The right panel (M: control, H: CAP) indicates that the H- and M-isotope clusters of the MT-ND6 peptides overlapped with the adjacent high-intensity peaks. This may explain why accurate quantification of H/M ratio of the peptide was hampered.

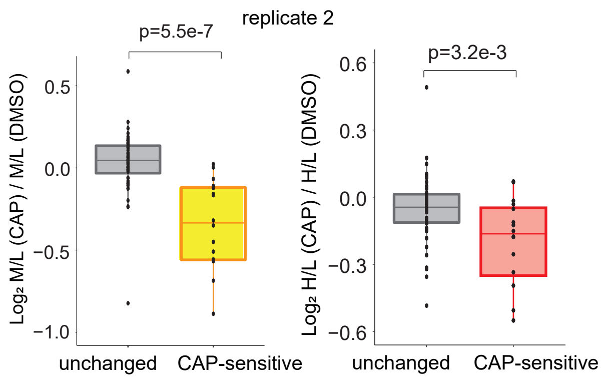

Figure S5: Validation of the pulse-chase experiments, related to Figure 5.

Effects of CAP on protein synthesis and degradation of nuclear-encoded subunits. A boxplot showing the degree of inhibition of protein synthesis of newly made M forms between CAP and DMSO treatments (left panel). A box plot showing the degree to which newly made H forms are degraded by CAP (right panel). “Unchanged” and “CAP-sensitive” represent nuclear-encoded subunits whose protein synthesis was unchanged and inhibited by CAP, respectively (see Figure 4). P-value was computed using the one-sided Wilcoxon rank-sum test.
